# Prognostic value of α-fetoprotein and des-γ-carboxy prothrombin responses in patients with hepatocellular carcinoma treated with transarterial chemoembolization

**DOI:** 10.1186/1471-2407-13-5

**Published:** 2013-01-03

**Authors:** Yong Kang Lee, Seung Up Kim, Do Young Kim, Sang Hoon Ahn, Kwang Hun Lee, Do Yun Lee, Kwang-Hyub Han, Chae Yoon Chon, Jun Yong Park

**Affiliations:** 1 Department of Internal Medicine, Yonsei University College of Medicine, 250 Seongsanno, Seodaemun–gu, Seoul 120-752, Korea; 2 Department of Radiology, Yonsei University College of Medicine, Seoul, Korea; 3 Liver Cancer Special Clinic, Yonsei University College of Medicine, Seoul, Korea; 4 Liver Cirrhosis Clinical Research Center, Seoul, Korea; 5 Brain Korea 21 Project for Medical Science, Seoul, Korea

**Keywords:** Alpha-fetoprotein, Des gamma carboxy prothrombin, Transarterial chemoembolization, Tumor marker response, Hepatocellular carcinoma, Prognosis, Survival

## Abstract

**Background/Aims:**

Alpha-fetoprotein (AFP) and des-gamma-carboxy prothrombin (DCP) have been used as diagnostic tools for hepatocellular carcinoma (HCC). However, prediction of outcome using AFP and DCP has not been elucidated. We investigated the clinical role of AFP and DCP as predictors of treatment outcome in patients with HCC undergoing trans-arterial chemoembolization (TACE).

**Methods:**

Between January 2003 and December 2005, we enrolled 115 treatment-naïve patients who received TACE as an initial treatment modality. An AFP or DCP response was defined as a reduction of more than 50% from the baseline level 1 month after TACE. Patients with AFP < 20 ng/mL or DCP < 20 mAU/mL were excluded.

**Results:**

The median age was 59 years and the male gender predominated (*n* = 81, 70.4%). AFP and DCP response was identified in 91 (79.1%) and 77 (66.9%) patients after TACE. Although progression-free survival (PFS) did not differ according to AFP response (*P* = 0.150), AFP responders showed significantly better overall survival (OS) than non-responders (34.9 *vs.* 13.2 months; *P* = 0.002). In contrast, DCP response did not influence either PFS or OS (all *P* > 0.05). Multivariate analyses showed that gamma-glutamyltranspeptidase and baseline AFP were predictors of PFS (all *P* < 0.05) and that male gender, the presence of liver cirrhosis, baseline DCP, number of measurable tumors and AFP response were independent predictors of OS (all *P* < 0.05).

**Conclusions:**

AFP response and higher baseline DCP level are significant predictors of OS in treatment-naïve patients with HCC receiving TACE who showed pretreatment elevation of both AFP and DCP.

## Background

Hepatocellular carcinoma (HCC) is the third most common cause of cancer-related death globally [1]. Unless HCC is diagnosed at an early stage when liver transplantation, hepatic resection, and radiofrequency ablation are feasible, a poor prognosis is expected due to the limited treatment options [2]. Although several treatment modalities have been applied to advanced HCC, [3,4] only trans-arterial chemoembolization (TACE) [5-10] and Sorafenib [11] exhibit survival benefit. Accordingly, TACE and Sorafenib are recommended for HCC in the intermediate and advanced Barcelona Clinic of Liver Cancer (BCLC) stages, respectively [12,13].

Alpha-fetoprotein (AFP) was first described about 40 years ago and is proposed as a screening and diagnostic tool for HCC [14-19]. Recently, the national comprehensive cancer network guidelines also proposed AFP as a alternative tool for diagnosing HCC [20]. Though there is no definitive evidence to support an absolute value of AFP cut-offs [21,22]. Moreover, several studies have reported that changes in AFP levels may predict treatment outcome [23-25]. A recent study showed a correlation between AFP response and radiologic response, time-to-progression (TTP), progression-free survival (PFS), and overall survival (OS) in patients treated with loco-regional therapies [26,27]. In contrast, controversies regarding the diagnostic role of des gamma carboxy prothrombin (DCP) remain. However, the clinical significance of DCP is emphasized in Asia [28]. Recently, its clinical usefulness was emphasized in a different ethnic group [17,29,30].

The primary aim of this study was to determine the clinical utility of AFP and DCP as predictors of treatment outcome in patients with HCC undergoing TACE.

## Patients and methods

### Patients

Between January 2003 and December 2005, a total of 270 patients underwent TACE as an initial treatment modality at Severance Hospital, Yonsei University College of Medicine, Seoul, Korea. Of these, we excluded 104 with an AFP level < 20 ng/mL [31] or DCP level < 20 mAU/mL, 47 without baseline or follow-up tumor markers, and four with curative resection after TACE. Finally, a total of 115 patients were recruited for statistical analysis and followed-up until December, 2009. The study protocol was approved by the independent institutional review board of our institute.

### Diagnosis of HCC

HCC was diagnosed based on the guidelines of American Association for the Study of Liver Diseases [32]. Briefly, patients were diagnosed with HCC if they had a tumor with a maximum diameter >2 cm, typical features of HCC on dynamic computed tomography (CT) (defined as hyperattenuation in the arterial phase and early washout in the portal phase), and an AFP >200 ng/ml [32]. If the maximum diameter of tumor was 1–2 cm, dynamic CT and magnetic resonance imaging (MRI) were performed. HCC was diagnosed if coincidental typical features of HCC were noted. If the tumor did not satisfy the above criteria, a biopsy was performed. When the tumor was <1 cm, ultrasonography was repeated after 3 months.

### Treatment and response-evaluation protocols

After diagnosis of HCC was confirmed, angiography was performed to identify the major arterial supply to HCC. TACE was conducted using a solution of 20–50 mg doxorubicin hydrochloride in a 5–20 ml mixed solution of lipiodol and contrast agent. Subsequently, embolization was performed using gelatin sponge particles after TACE. After TACE, occlusion of target vessels and absence of additional tumor blood supply was confirmed.

Radiological response evaluation using CT or MRI and tumor marker response were evaluated 3–4 weeks after TACE. If viable tumor remained at CT or MRI, TACE was repeated. After 2–3 consecutive TACE sessions, at the time of best radiologic response, radiological and tumor marker responses were evaluated. If compact lipiodolization was noted and no viable HCC was identified, than radiologic evaluation and tumor marker measurement were done within 3 months to assess HCC recurrence. If progressive disease was identified, other treatment modalities were considered.

### Tumor marker response evaluation

Baseline AFP and DCP levels were determined before TACE. Follow up tumor marker were checked every 3 ~ 4 weeks after TACE concurrent with CT or MRI. To analyze tumor marker response, we determined AFP and DCP levels when the best radiologic response was reached after 2–3 consecutive sessions of TACE. Tumor marker response evaluation was performed when complete remission is observed after consecutive session of TACE in most of subjects, but tumor marker response also analyzed within subject who did not get complete remission. In these patients, tumor marker checked just before last TACE concluded as progressive diseases by mRECIST criteria was used. AFP/DCP levels were measured using a microparticle enzyme immunoassay (AFP, Bayer, Leverkusen, Germany; DCP, Sanko Junyaku Co., Tokyo, Japan). AFP response was defined as a reduction in AFP level of more than 50% from baseline, according to a previous study [27]. Similarly, DCP response was defined as a reduction in DCP level of more than 50% from baseline.

### Radiologic response evaluation

Radiologic tumor response was evaluated using the WHO and modified RECIST criteria by CT or MRI. For the WHO criteria, minimum cross product was compared to baseline cross product for calculating change in size. Complete response (CR) was defined as complete disappearance; partial response (PR) was defined as 50% decrease; progressive disease (PD) was defined as a more than 25% increase in the cross product from maximum decrease. All other findings were defined as stable disease (SD). In the mRECIST criteria, length of the major axis of a viable tumor was compared to baseline for calculating change in size. CR was taken as the absence of any enhancing tissue, PR as a 30% decrease in enhancing tissue, and SD as a less than 20% decrease.

### Calculation of progression-free and overall survival

The progression-free survival (PFS) time is defined as the time from the TACE start date to the first observation of tumor progression confirmed by mRECIST criteria or death due to any cause. To account for the fact that some patients dropped out for reasons other than tumor progression, PFS for these patients was censored at the time of last follow-up. The overall survival (OS) was calculated from the time of study entry to death. If the patient had no recorded date of death or was still alive at the time of the analysis, OS for these patients was censored at the date that the patient was last seen alive.

### Statistical analysis

An independent *t*-test or Mann–Whitney *U* test was used to compare continuous variables, and a chi-square or Fisher’s exact test was used to compare categorical variables between two groups (AFP responder *vs.* AFP non-responder and DCP responder *vs.* DCP non-responder). AFP and DCP response together with conventional clinical variables at the time of entry to the study were used to identify independent factors that influence PFS and OS via the Cox proportional hazards model. Hazard ratio (HR) and the corresponding 95% confidence intervals (CI) were also indicated. Age, sex, etiology, anti-viral therapy, HBV-DNA positivity, Child Pugh classification, liver cirrhosis, GGT, baseline AFP and DCP, AFP response, DCP responce, BCLC stage, number of tumor, size of tumor, WHO response and mRECIST response were included in univariate regression test on PFS and OS. Gender, Child-Pugh Classification, GGT, AFP, DCP, number and size of tumor, BCLC stage were included in multivariate regression test on PFS. Gender, Cirrhosis, GGT, AFP response, Baseline DCP, number and size of tumor were included on OS analysis. PFS and OS were calculated using the Kaplan–Meier method and compared using a log rank test. Statistical analyses were performed using SPSS ver. 11.0 software (SPSS Inc., Chicago, Ill).

## Results

### Baseline characteristics

Baseline characteristics of the 115 patients are described in Table 1. The median age was 59 years and male gender predominated (*n* = 81, 70.4%). Hepatitis B virus was the most common etiology of HCC (*n* = 80, 69.6%). The median baseline AFP and DCP were 296.7 ng/mL and 231 mAU/mL, respectively. Median follow up was 26 (range, 1.2–75.9) months.

**Table 1:** Baseline characteristics

**Variables**	**AFP**			***P *value**	**DCP**		***P *value**
	**All patients**	**AFP responder**	**AFP non-responder**		**DCP responder**	**DCP non-responder**	
	**(n = 115)**	**(n = 91, 79.1%)**	**(n = 24, 20.9%)**		**(n = 77, 66.9%)**	**(n = 38, 33.1%)**	
Age, years	59 (37 - 78)	59 (37 - 78)	59 (39 - 75)	0.783	59 (37 - 78)	59.9 (40 - 77)	0.661
Male	81 (70.4)	63 (69.2)	18 (75.0)	0.802	53 (68.8)	28 (73.7)	0.668
Etiology							
HBV/ HCV/ Others	80 (69.5)/ 18 (15.7)/ 17 (14.8)	63 (69.2)/ 15 (16.5) /13 (14.3)	17 (70.8)/ 3 (12.5)/ 4 (16.7)	0.883	53 (68.8)/ 13 (16.9)/ 11 (14.3)	27 (71.0)/ 5 (13.2)/ 6 (15.8)	0.908
Child-Pugh, A *vs*. B	108 (93.9)/ 7 (6.1)	89 (97.8)/ 2 (2.2)	19 (79.2)/ 5 (20.8)	0.004	74 (96.1) / 3 (3.9)	34 (89.5)/ 4(10.5)	0.217
Liver cirrhosis	72 (62.6)/ 43 (37.4)	56 (61.5)/ 35 (38.5)	16 (66.7)/ 8(33.3)	0.813	46 (59.7) / 31 (40.3)	26 (68.4)/ 12(31.6)	0.417
Biochemical Variables							
Platelet, 10^3^/uL	127 (38 - 414)	134 (38 - 332)	108 (64 - 414)	0.585	123 (38 - 332)	133.5 (60-414)	0.467
ALT, IU/L	38 (12 - 315)	39 (12 - 315)	38 (18 - 116)	0.088	39 (12 - 257)	37 (18 - 315)	0.561
Bilirubin, mg/dL	0.7 (0.2 - 13.4)	0.7 (0.2 - 3.0)	0.6 (0.3 - 13.4)	0.405	0.7 (0.2 - 3.0)	0.6 (0.2-13.4)	0.462
AFP, ng/mL	296.7 (24.23 - 83000)	320.95 (24.23 - 50000)	142.96 (26 - 83000)	0.496	320.95 (24.23 - 83000)	280.08 (25.22 - 50000)	0.774
DCP, mAU/mL	231 (20 - 2000)	231 (20 - 2000)	228.5 (23 - 2000)	0.304	276 (20 - 2000)	62.5 (20 - 2000)	0.464
Number of tumor^a^							
1/ 2/ 3/ >4	60 (52.2)/ 18 (15.7)/ 9 (7.8)/ 28 (24.3)	13 (54.2)/ 5 (20.8)/ 2 (8.3)/ 4 (16.7)	47 (51,6)/ 13 (14.3)/ 7 (7.7)/ 24 (26.4)	0.732	35(45.4)/ 13 (16.9)/ 6 (7.8)/ 23 (29.9)	25 (65,8)/ 5 (13.2)/ 3(8.0)/ 5(13.2)	0.160
Size of tumor, mm^b^	47 (10 - 160)	47 (10 - 160)	45.5 (10 - 151)	0.531	49 (10 - 160)	36 (10 - 160)	0.473
BCLC stage							
A/ B/ C	62 (53.9)/ 49 (42.6)/ 4 (3.5)	47 (51.6)/ 40 (44.0)/ 4 (4.4)	15 (62.5)/ 9 (37.5)/ 0 (0.0)	0.484	40 (51.9)/ 36 (46.8)/ 1 (1.3)	22 (57.9)/ 13 (34.2)/ 3 (7.9)	0.117
TNM stage of LCSGJ							
I/ II/ III/ IVa	21 (18.3)/ 40 (34.8)/	19 (20.9)/ 29 (31.9)/	2 (8.3)/ 11 (45.8)/	0.412	9 (11.7)/ 31 (40.3)/	12 (31.6)/ 9 (23.7)/	0.027
	42 (36.5)/ 12 (10.4)	34 (37.4)/ 9 (9.9)	8 (33.3)/ 3 (1.5)		27 (35.1)/ 10 (13.0)	15 (39.5)/ 2 (5.3)	

Variables are expressed as n (%) or median (range).

^a^number of measurable lesion, ^b^total size of measurable lesion.

AFP, alpha-fetoprotein; DCP, des gamma carboxy prothrombin; HBV, hepatitis B-virus; HCV, hepatitis C-virus; ALT, alanine aminotransferase; BCLC, Barcelona Clinic Liver Cancer; TNM, Tumor-Node-Metastasis; LCSGJ, Liver Cancer Study Group of Japan.

### Tumor marker responses

After TACE, 91 (79.1%) patients showed AFP response and 24 (20.9%) were non-responders (Table 1). Baseline characteristics were similar between AFP responders and non-responders, except for the significantly higher proportion of Child–Pugh class A in AFP responders than non-responders (97.8 *vs.* 79.2%; *P* = 0.004). In contrast, 77 (66.9%) patients showed a DCP response, and 38 (33.1%) did not (Table 1). DCP non-responders showed a significantly higher proportion of solitary HCC (65.8 *vs.* 45.4%; *P* = 0.048).

### Objective responses after TACE and correlation with tumor marker responses

Table 2 shows objective responses after TACE using WHO and mRECIST criteria and their correlations with AFP and DCP responses. Neither AFP nor DCP responses showed a significant correlation with radiological response evaluated using the WHO criteria. In contrast, AFP, but not DCP, response was significantly correlated with radiological response evaluated using the mRECIST criteria (*P* = 0.045).

**Table 2:** Objective responses after TACE and correlation with tumor marker responses

**Radiologic response**	**AFP response**		***P *value**	**DCP response**		***P *value**
	**AFP responder**	**AFP non-responder**		**DCP responder**	**DCP non-responder**	
	**(n = 91, 79.1%)**	**(n = 24, 20.9%)**		**(n = 77, 66.9%)**	**(n = 38, 33.1%)**	
WHO criteria			0.196			0.308
Complete response	0 (0)	0 (0)		0 (0)	0 (0)	
Partial response	17 (18.7)	3 (12.5)		11 (14.3)	9 (23.7)	
Stable disease	71 (78.0)	18 (75.0)		63 (81.8)	26 (68.4)	
Progressive disease	3 (3.3)	3 (12.5)		3 (3.9)	3 (7.9)	
mRECIST criteria						
Complete response	70 (76.9)	13 (54.2)	0.045	55 (71.4)	28 (73.7)	0.298
Partial response	11 (12.1)	3 (12.5)		12 (15.6)	2 (5.3)	
Stable disease	3 (3.3)	3 (12.5)		4 (5.2)	2 (5.3)	
Progressive disease	7 (7.7)	5 (20.8)		6 (7.8)	6 (15.7)	

Variables are expressed as n (%).

AFP, alpha-fetoprotein; DCP, des gamma carboxy prothrombin; WHO, World health organization; mRECIST, modified Response Evaluation Criteria in Solid Tumor.

### Independent predictors for PFS and OS

**Figure 1:**
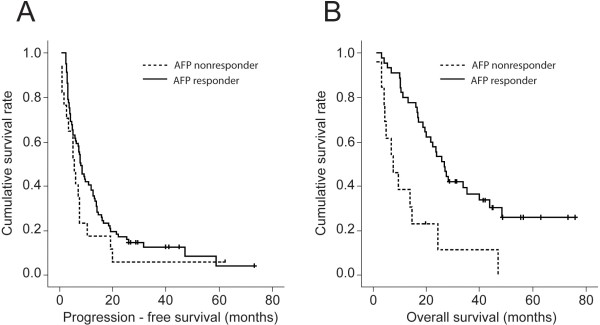
**Progression-free (PFS) and overall survival (OS) curves of AFP responder and non-responder. **PFS was similar between AFP responders and non-responders (8.0 *vs. *5.5 months; log rank test, *P* = 0.150; **(A)**) whereas OS were significantly better in AFP responder than non-responder. (34.9 *vs.* 13.2 months; log rank test, *P* = 0.020; **(B)**).

**Figure 2:**
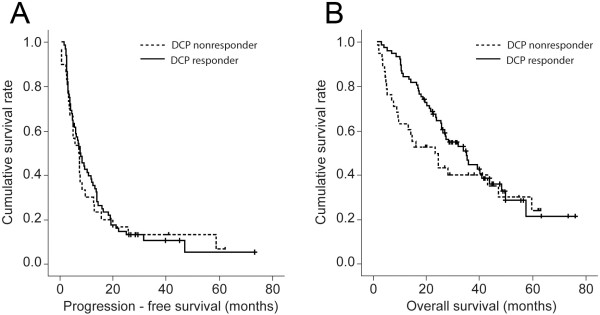
**Progression-free survival (PFS) and overall survival (OS) curves of DCP responder and non-responder. **Both PFS and OS were not significantly different between DCP responder and non-responder (7.8 *vs.*7.3 months; log rank test, *P* = 0.755 for PFS **(A) **and 34.9 *vs. *23.2 months; log rank test, *P* = 0.203 for OS **(B)**).

Univariate and multivariate analyses were used to identify independent predictors of PFS and OS (Table 3). Regarding PFS prediction, gamma glutamyltranspeptidase (GGT) (HR, 1.003; 95% CI 1.001–1.004; *P* = 0.012) and baseline AFP (HR 1.000; 95% CI 1.000–1.001; *P* = 0.049) were identified as independent predictors. Regarding OS prediction, male gender (HR, 2.119; 95% CI, 1.040–4.320; *P* = 0.039), the presence of cirrhosis (HR, 2.319; 95% CI, 1.281–4.201; *P* = 0.005), baseline DCP level (HR, 1.000; 95% CI, 1.000–1.001; *P* = 0.028), number of tumors(HR, 1.443; 95% CI, 1.035-2.011; *P* = 0.030) and AFP response (HR, 0.276; 95% CI; 0.147–0.518; *P* < 0.001) were independent predictors of OS. The Kaplan–Meier curves of PFS and OS according to AFP and DCP responses are indicated in Figures 1 and 2.

**Table 3:** Independent predictors for progression-free and overall survival

**Variables**	**Progression-free survival**				**Overall survival**			
	**Univariate**	**Multivariate**			**Univariate**	**Multivariate**		
	***P *value**	***P *value**	**HR**	**95%CI**	***P *value**	***P *value**	**HR**	**95%CI**
Age, years	0.770		-	-	0.578		-	-
Male	0.028	0.443	-	-	0.013	0.039	2.119	1.040-4.320
Etiology								
Viral *vs.* others	0.252		-	-	0.916		-	-
Anti-viral therapy	0.325		-	-	0.047	0.453	-	-
HBV-DNA positivity	0.165		-	-	0.231		-	-
HBeAg positivity	0.234		-	-	0.324		-	-
Child-Pugh class, A *vs*. B	0.040	0.832	-	-	0.787		-	-
Liver cirrhosis	0.101		-	-	0.002	0.005	2.319	1.281-4.201
GGT	0.001	0.012	1.003	1.001-1.004	0.014	0.068	-	-
Tumor marker								
Baseline AFP	0.008	0.049	1.000	1.000-1.001	0.486		-	-
Baseline DCP	0.039	0.686	-	-	0.001	0.028	1.000	1.000-1.001
AFP response	0.155		-	-	0.023	<0.001	0.276	0.147-0.518
DCP response	0.756		-	-	0.205		-	-
BCLC stage, A *vs*. ≥B	0.002	0.409	-	-	0.003	0.502	-	-
Number of tumor^a^	0.001	0.373	-	-	<0.001	0.030	1.443	1.035-2.011
Size of Tumor^b^	0.021	0.927	-	-	<0.001	0.085	-	-
WHO response								
CR + PR + SD *vs*. PD	0.419		-	-	0.945		-	-
mRECIST								
CR + PR + SD *vs*. PD	0.107		-	-	0.548		-	-

^a^number of measurable lesion, ^b^total size of measurable lesion.

HR, hazard ratio; CI, confidence interval; AFP, alpha-fetoprotein; DCP, des gamma carboxy prothrombin; BCLC, Barcelona Clinic Liver Cancer.

Reference value: others, Child-Pugh class B, BCLC stage ≥ B, PD with WHO criteria, and PD with mRECIST criteria.

### DCP response within higher DCP levels

DCP response did not show statistical significance for prediction of PFS or OS after TACE. However, when we analyzed a subgroup of patients with a baseline DCP level > 200 mAU/ml [31], a significant correlation between DCP response and OS emerged (median 26.7 months in DCP responders *vs.* 7.5 months in DCP non-responders; log rank test *P* = 0.002). Thus, application of DCP response to prediction of treatment response and the corresponding optimal cutoff values of baseline DCP level to enhance the treatment-outcome predictability of DCP response should be further investigated.

### Combined serological endpoint

Sub-analysis was done to find out whether combined serological endpoint (AFP and/or DCP responses) would improve it prognostication [33]. AFP and/or DCP responders were stratified into combined tumor marker responders (cTM responders, *n* = 99) whereas subjects without AFP and DCP responses were stratified into cTM non-responders (*n* =16). PFS was similar between cTM responders and non-responders (15.1 *vs.* 10.5 months; log rank test, *P* = 0.259) whereas OS was significantly longer in cTM responders than non-responders (39.0 *vs.* 21.5 months; log rank test, *P* = 0.011). In addition, cTM response was selected as one of independent predictors of OS (HR 0.312; 95% CI 0.150-0.649; *P* = 0.002), together with tumor size and cirrhosis. However, cTM response did not predict PFS independently.

### Discordance between cTM response and radiologic response

We divided the study population according to cTM and radiological responses. Definition of cTM response described at combined serological endpoint. Similarly, radiologic responders were defined as patients with CR/PR/SD and radiologic non-responders as those with PD using the mRECIST criteria. Using these definitions, eight (6.9%) cTM responders experienced a radiologic non-response and 12 (10.4%) cTM non-responders experienced radiologic responses; these were categorized as discordant.

Baseline albumin level was identified as an independent predictor of discordance between cTM response and radiologic response (HR, 2.747; 95% CI, 1.266–2.963; *P* = 0.011). Analysis of the discordant group (*n* = 20) revealed that the PFS and OS of eight cTM responders with radiologic non-response was not statistically different from that of 12 cTM non-responders with radiologic response (5.1 *vs.* 5.1 months; *P* = 0.828 for PFS and 33.8 *vs.* 7.5 months; *P* = 0.354 for OS) [Additional file 1].

## Discussion

Tumor response evaluation using CT or MRI after systemic chemotherapy or loco-regional treatment is widely used to decide whether to treat further or change treatment strategies. However, high cost, radiation hazard, and associated cancer risks are drawbacks to these response evaluation modalities, [34] whereas tumor marker evaluation after anti-cancer treatment is rapid, easier to estimate, and less expensive. Thus, the clinical implications of tumor response evaluation using tumor markers have been continuously investigated [35-38].

This study also showed that AFP response can be an independent predictor of OS in patients undergoing TACE as the first treatment modality, whereas radiologic response was not significant in predicting PFS and OS. Several previous studies have also proposed the clinical usefulness of AFP response. Riaz et al. [27] proposed AFP response after loco-regional therapy as an ancillary method of assessing tumor response and survival, as well as an early objective screening tool for progression by imaging. Furthermore, the supplementary role of AFP response for tumor response evaluation and prediction of better survival and extrahepatic metastasis in patients undergoing hepatic arterial infusional chemotherapy, localized concurrent chemoradiation therapy, and anti-angiogenic treatment such as Sorafenib and Bevacizumab has been proposed [26,36,37]. However, to our disappointment, AFP response didn’t show its novel value of prediction in PFS of HCC patients. It might be caused by that all subjects did not have the same follow-up period and timing of assessment scan, furthermore definition of PFS might be subject to bias. So it could be a limitation of our study.

The baseline AFP level in our study also showed statistical significance for predicting PFS in a multivariate analysis (*P* = 0.035). Similarly, the significance of the prognostic role of baseline AFP level has been accepted in previous studies and several staging systems [39,40]. In contrast, baseline DCP level was significantly associated with OS in our study. Several other studies investigated the usefulness of baseline DCP level, and demonstrated that higher baseline DCP level was significantly correlated with poor prognosis in HBV-related HCC and with a higher risk of recurrence after curative treatment [35,41]. Although the reasons why HCC with a higher baseline DCP level is related to poor prognosis remain uncertain, it may in part be due to the fact that the higher baseline DCP level is significantly associated with the presence of vascular invasion, intra-hepatic metastasis, tumor size, Tumor-Node-Metastasis (TNM) stage, and a high frequency of tumor recurrence [42].

When we consider that only viable HCC can produce AFP and that the mRECIST criteria were developed to evaluate the amount of viable HCC, it is not surprising that AFP response showed a significant correlation to tumor response evaluated by mRECIST criteria. Although DCP is also produced by viable HCC, DCP response was not correlated with mRECIST response. This may be because, in contrast to AFP, DCP can be produced in surrounding non-cancer tissues after being stimulated by HCC [42], which might have attenuated the correlation between DCP response and radiologic tumor response by the mRECIST criteria. This also supports our result that DCP response was not significant for predicting PFS or OS after TACE. Because the WHO criteria do not consider specific situations (such as necrosis and the viable portion of HCC) neither the AFP nor DCP responses were correlated with radiologic response evaluation by the WHO criteria.

We further analyzed patients showing discordant outcomes assessed by cTM response and radiologic response, because in this situation physicians must decide whether to maintain the current treatment modality or change it. Although baseline albumin level was identified as a predictor of discordance between cTM response and radiologic response, there was no statistical difference in OS between cTM responders with radiologic non-response and those with cTM non-response but radiologic response (33.8 *vs.* 7.5 months; *P* = 0.354). Because a longer OS was noted in patients with cTM response and radiologic non-response in spite of the lack of statistical significance and because the clinical significance of the cTM response might have been attenuated due to the small discordant sub-population, a larger study is needed to reveal the role of cTM response in these cases. In the same context, if the role of cTM response in discordant cases can be demonstrated, cTM non-responders who experience radiologic response may need to have their disease progression monitored closely.

Interestingly, baseline GGT level was identified as an independent prognostic factor of PFS, similar to a recent study that proposed that GGT level is an important prognostic factor in patients with BCLC-intermediate HCC treated with TACE [43]. Considering that GGT level is associated with oxidative stress and resistance to platinum drugs [44,45], the prognostic value of GGT levels for discriminating subgroups with different risks of disease progression or mortality should be determined.

This study has limitations. First, this was a retrospective study with a relatively small sample size. Also, some study subjects were included in article published recently and analyzed about prognostic value of AFP and DCP by the same institute [46]. However, the aim of this study is clearly different from previous one focusing on the prognostic value of baseline AFP and DCP in all patients with treatment-naïve HCC. In the present study, we focused on the prognostic value of the responses of AFP and DCP by TACE. Second, we excluded patients with low baseline AFP or DCP levels before TACE. Our results might not apply to patients with low baseline AFP or DCP levels. However, because we simply investigated the prognostic values of AFP and DCP response simultaneously, we inevitably selected only patients with elevated AFP and DCP in spite of a potential selection bias. Third, because AFP can be confounded by hepatic necroinflammation, the predictability of the AFP response might have been influenced. AFP response could be poor in patients with persistently elevated ALT after TACE. Furthermore, patients with persistently elevated ALT are reportedly associated with poor OS due to more rapid impairment of the liver function. Physicians should be cautious in applying baseline AFP or AFP response to patients suffering from active hepatitis or to those without elevated baseline AFP and DCP levels. Finally, considering that TACE was introduced as a palliative treatment for intermediate-stage HCC, many HCC patients with BCLC stage A in our study who were indicated for curative treatment underwent TACE. For several reasons, these patients decide to take TACE. Some of them refused surgical resection and others are unable to surgical resection because of their medical condition such as old age(>70 yrs) and poor general conditions (ECOG PS ≥ 2 and/or cachexia). Furthermore, sub-segmental TACE using iodized oil and a gelatin sponge remains an important therapeutic option for patients with HCC in Japan [47]. This is not surprising, because sub-segmental TACE as a curative treatment modality has become widely accepted in Korea as well as in Japan.

## Conclusions

The present study demonstrated the clinical importance of AFP response and baseline DCP level for predicting OS in treatment-naïve patients with HCC undergoing TACE as an initial treatment modality. In the future, a treatment algorithm incorporating tumor marker response and radiologic response, which compensate for each other’s drawbacks, should be established to more accurately predict treatment outcomes after TACE.

## Abbreviations

HCC: Hepatocellular carcinoma; TACE: Trans-arterial chemoembolization; BCLC: Barcelona clinic liver cancer; AFP: Alpha-fetoprotein; TTP: Time to progression; PFS: Progression free survival; OS: Overall survival; DCP: Des gamma carboxy prothrombin; WHO: World Health Organization; RECIST: Response evaluation criteria in solid tumor; mRECIST: Modified response evaluation criteria in solid tumor; CT: Computed tomography; MRI: Magnetic resonance image; CR: Complete remission; PR: Partial remission; PD: Progressive disease; SD: Stable disease; HR: Hazard ratio; CI: Confidence interval; GGT: Gamma glutamyltranspeptidase; HBV: Hepatitis B-virus; HCV: Hepatitis C-virus; ALT: Alanine aminotransferase; HBeAg: Hepatitis B e antigen; HBV-DNA: Hepatitis B virus-deoxyribonucleic acid; TNM: Tumor-Node-Metastasis; cTM: Combined tumor marker; LCSGJ: Liver cancer study group of Japan; ECOG PS: Eastern Cooperative Oncology Group performance status.

## Competing interest

The authors declare that they have no competing interests.

## Authors’ contributions

Guarantor of the article: Jun Yong Park, MD, Specific author contributions: YKL and SUK organized the protocol, collected and analyzed the data, and prepared the manuscript. DYK, SHA, KHH, and CYC recruited the patients and assisted article preparation. DYL carried out transarterial chemoembolization in HCC. JYP had the original idea for the study, was involved in the study design, and assisted article preparation. All authors read and approved the final manuscript.

## Pre-publication history

The pre-publication history for this paper can be accessed here:

http://www.biomedcentral.com/1471-2407/13/5/prepub

## Supplementary Material

Additional file 1 **Table S1. **Independent predictors between cTM responder with Radiologic non-responder ( *n *= 8 ) and cTM non-responder with radiologic responder ( *n* = 12 ). **Figure S1. **Progression-free survival (PFS) and overall survival (OS) curves of TM responder with radiologic non-responder and TM non-responder with radiologic non-responder. Both PFS and OS were not significantly different between TM responder with radiologic non-responder and TM non-responder with radiologic non-responder (5.1 *vs*. 5.1 months; log rank test, *P*=0.828 for PFS (A) and 33.8 *vs. *7.5 months; log rank test, *P*=0.354 for OS (B)). **Figure S2.** Progression-free survival (PFS) and overall survival (OS) curves of cTM responder with radiologic responder and cTM non-responder with radiologic non-responder. PFS was similar between cTM responder with radiologic responder and cTM non-responder with radiologic non-responder (19.0 *vs*. 6.2 months; log rank test, *P*=0.065 for PFS (A)) whereas OS were significantly better in cTM responder with radiologic responder than cTM non-responder with radiologic non-responder (39.2 *vs. *12.8 months; log rank test, *P*=0.031 for OS (B)). Click here for file
