# Supplementary material for: Prognostic value of α-fetoprotein and des-γ-carboxy prothrombin responses in patients with hepatocellular carcinoma treated with transarterial chemoembolization
Source: BMC Cancer. 2013 Jan 3;13:5. doi: 10.1186/1471-2407-13-5 (PMC3545962; doi:10.1186/1471-2407-13-5)
Supplement: Additional file 1 — Table S1. Independent predictors between cTM responder with Radiologic non-responder ( n = 8 ) and cTM non-responder with radiologic responder ( n = 12 ). Figure S1. Progression-free survival (PFS) and overall survival (OS) curves of TM responder with radiologic non-responder and TM non-responder with radiologic non-responder. Both PFS and OS were not significantly different between TM responder with radiologic non-responder and TM non-responder with radiologic non-responder (5.1 vs. 5.1 months; log rank test, P=0.828 for PFS (A) and 33.8 vs. 7.5 months; log rank test, P=0.354 for OS (B)). Figure S2. Progression-free survival (PFS) and overall survival (OS) curves of cTM responder with radiologic responder and cTM non-responder with radiologic non-responder. PFS was similar between cTM responder with radiologic responder and cTM non-responder with radiologic non-responder (19.0 vs. 6.2 months; log rank test, P=0.065 for PFS (A)) whereas OS were significantly better in cTM responder with radiologic responder than cTM non-responder with radiologic non-responder (39.2 vs. 12.8 months; log rank test, P=0.031 for OS (B)). [file 1471-2407-13-5-S1.docx]

| **Table S1.** Independent predictors between cTM responder with Radiologic non-responder ( *n* = 8 ) and cTM non-responder with radiologic responder ( *n* = 12 ). | | | | | |
| --- | --- | --- | --- | --- | --- |
| Variables | Binary regression test | | | | |
|  | Univariate |  | Multivariate | | |
|  | *P* value |  | *P* value | HR | 95% CI |
| Age, years | 0.198 |  |  |  |  |
| Male | 0.264 |  |  |  |  |
| Etiology |  |  |  |  |  |
| Viral *vs.* others | 0.976 |  |  |  |  |
| Child-Pugh class |  |  |  |  |  |
| A/ B | 0.810 |  |  |  |  |
| Liver cirrhosis | 0.454 |  |  |  |  |
| Tumor marker |  |  |  |  |  |
| Baseline AFP | 0.828 |  |  |  |  |
| Baseline PIVKA-II | 0.254 |  |  |  |  |
| BCLC stage |  |  |  |  |  |
| A *vs*. ≥ B | 0.915 |  |  |  |  |
| Albumin | 0.011 |  | 0.011 | 2.747 | 1.266-5.963 |

**
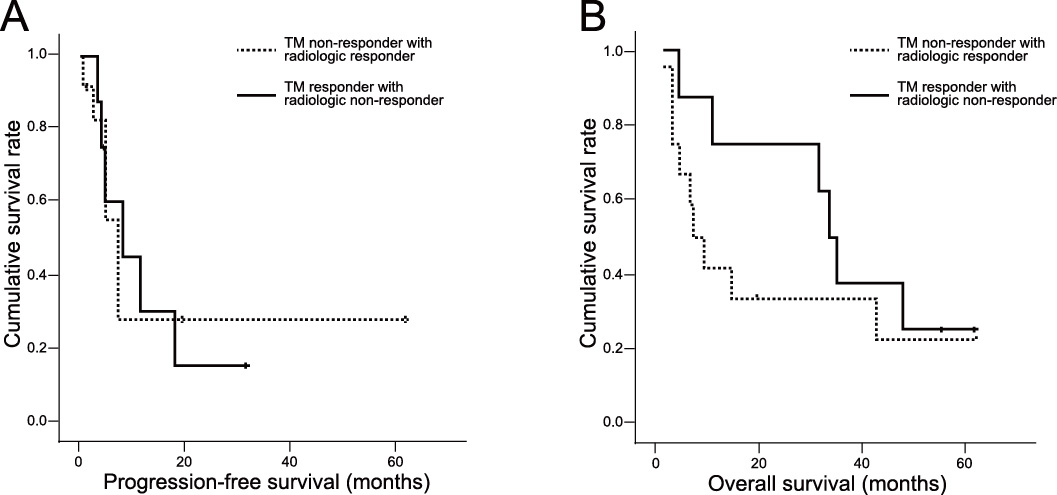
**

**Figure S1**. Progression-free survival (PFS) and overall survival (OS) curves of TM responder with radiologic non-responder and TM non-responder with radiologic non-responder. Both PFS and OS were not significantly different between TM responder with radiologic non-responder and TM non-responder with radiologic non-responder (5.1 *vs*. 5.1 months; log rank test, *P*=0.828 for PFS (A) and 33.8 *vs.* 7.5 months; log rank test, *P*=0.354 for OS (B))

**Figure S2**. Progression-free survival (PFS) and overall survival (OS) curves of cTM responder with radiologic responder and cTM non-responder with radiologic non-responder. PFS was similar between cTM responder with radiologic responder and cTM non-responder with radiologic non-responder (19.0 *vs*. 6.2 months; log rank test, *P*=0.065 for PFS (A)) whereas OS were significantly better in cTM responder with radiologic responder than cTM non-responder with radiologic non-responder (39.2 *vs.* 12.8 months; log rank test, *P*=0.031 for OS (B))
